# Synthesis and Characterization of Tin(IV) Oxide Obtained by Chemical Vapor Deposition Method

**DOI:** 10.1186/s11671-016-1547-x

**Published:** 2016-07-26

**Authors:** Svitlana V. Nagirnyak, Victoriya A. Lutz, Tatiana A. Dontsova, Igor M. Astrelin

**Affiliations:** Department of Chemistry, National Technical University of Ukraine “KPI”, Kyiv, 03056 Ukraine

**Keywords:** Tin(IV) oxide, Tin(II) oxalate, CVD method, X-ray diffraction, Bandgap

## Abstract

The effect of precursors on the characteristics of tin oxide obtained by chemical vapor deposition (CVD) method was investigated. The synthesis of nanosized tin(IV) oxide was carried out with the use of two different precursors: tin(II) oxalate obtained using tin chloride(II) and oxalic acid; tin(II) oxalate obtained using tin chloride(II); and ammonium oxalate. The synthesized tin(IV) oxide samples were studied by electron microscopy, X-ray diffraction and optical spectra. The lattice parameters of tin(IV) oxide samples were defined, the bandgap of samples were calculated.

## Background

Metal oxides are the basis of modern diverse smart and functional materials and devices because physical and chemical properties of these oxides can be tuned.

Functional properties of metal oxides depends on many chemical and structural characteristics such as chemical composition, various kinds of deficiencies, morphology, particle size, surface-to-volume ratio, etc. By varying either of these characteristics, the electrical, optical, magnetic, and chemical properties can be regulated, giving the possibility of fabricating smart devices. Such unique characteristics make oxides the most diverse class of materials, with properties covering almost all aspects of materials science and physics in areas such as semiconductivity, superconductivity, ferroelectricity, and magnetism [1–4].

It is known that the reversible chemisorption of reactive gases on the surface of the oxide semiconductor is accompanied by reversible changes in conductivity. This makes semiconductors the most attractive materials for the manufacture photosensitive electronic converters based on them. Conductivity of semiconducting oxides caused by deviations from stoichiometry and also defects such as interstitial cation or anion vacancies. Depending on type of determinate impurity (electron acceptor or electron donor) and conduction type (*n*- or *p*-type), the resistance of the sensitive layer of the sensor is increased or decreased. Oxidizing gases or electron acceptors such as NO_2_ produce a decrease in the conductance of *n*-type semiconducting materials (i.e., electrons are the major carriers, such as ZnO, SnO_2_, In_2_O_3_) and an increase in the conductance of *p*-type semiconducting materials (i.e., holes are the major carriers, such as CuO); reducing gases or electron donors such as H_2_S, CO, H_2_ and water vapor act in a reverse manner [5, 6].

Metal oxides SnO_2_, ZnO, In_2_O_3_, and CdO are wide-bandgap *n*-type semiconductors and the most frequently used as a sensitive material for the gas sensors. They belong to a class of transparent conductive oxides due to a number of unique functional properties, of which the most important are the electrical conductivity, the visibility in a wide spectral range, and high reactivity of the surface [7, 8].

Metal oxide-based gas sensors are widely used due to its high sensitivity to harmful for human health or hazardous gases (such as CO, NO, NO_2_, H_2_, etc.) in conjunction with easy fabrication methods and low manufacturing costs. Tin(II) oxide is the promising sensor material among a wide set of semiconducting metal oxides [9–11]. It is known that nanocrystalline materials characterized the largest values of sensor response due to high surface-to-volume ratio and, therefore, higher absorption capacity [6].

To obtain nanocrystalline, SnO_2_ uses different methods: sol-gel method [12], chemical vapor deposition [13], hydrothermal [14], thermal evaporation [15]. Among a large number of approach methods of chemical vapor deposition (CVD), which is implemented of vapor-liquid-solid mechanism (VLS), deserves special attention. This method allows obtaining particles of very diverse morphology with a high degree of crystallinity [1, 16, 17]. In the articles [18–20], SnO_2_ nanowires and nanoribbons (doped and pure) have been successfully synthesized using such precursors as Sn and SnO_2_ powders. Also known to use other precursors for synthesis of SnO_2_ nanowires are SnO powder, and a mixture of carbon powder and SnO_2_ powder. However, from our point of view, it is interesting to research also other precursors, as has long been known that precursors have a significant impact on the final physicochemical properties of materials. In this paper, we investigate the effect of new precursor SnC_2_O_4_ (obtained from different reagents) on the characteristics of tin oxide obtained by CVD.

## Methods of synthesis

Tin(II) oxalate was obtained by sol-gel method from different precursors: in the first case tin chloride(II) and oxalic acid; in the second case – tin chloride(II) and ammonium oxalate. In both cases, hot oxalic acid (ammonium oxalate) solution was added to hot aqueous solutions of SnCl_2_ · 2H_2_O in a molar ratio of 1:1.5, respectively. The resulting solution was cooled. The precipitate formed was filtered, washed with distilled water while ions Cl^−^ detected by reaction with AgNO_3_ and dried in an oven at 378 K for 2 h. Thus, there were two obtained samples of tin oxalate: sample A – using oxalic acid and sample B – using ammonium oxalate (Table 1).

**Table 1:** Obtained SnC_2_O_4_ samples

Sample	Precursors	Treatment temperature, K
Sample A	Tin chloride(II); oxalic acid	378
Sample B	Tin chloride(II); ammonium oxalate	378

For tin(IV) oxide weighed tin(II) oxalate was loaded in an alumina boat, which was placed at the center of a quartz tube in a horizontal-type furnace. The furnace was heated to 1123, 1223, and 1323 K and kept in an inert atmosphere for 1 h. The inert atmosphere was implemented by nitrogen with 0.005 % oxygen impurity.

The overall reaction of tin(II) oxalate decomposition:

$$ {\mathrm{SnC}}_2{\mathrm{O}}_4\to\ {\mathrm{SnO}}_2 + 2\mathrm{C}\mathrm{O} $$

Obtained SnO_2_ samples (Table 2) were analyzed by electron microscopy, X-ray diffraction and spectrophotometrically.

**Table 2:** Obtained SnO_2_ samples

Sample	Precursors	Treatment temperature, K
Sample 1	Sample A	1123
Sample 2	Sample A	1223
Sample 3	Sample A	1323
Sample 4	Sample B	1123
Sample 5	Sample B	1223
Sample 6	Sample B	1323

## Results and Discussion

### Electron Microscopy

The particle sizes of the obtained samples were determined with a transmission electron microscope TEM 100-01.

**Fig. 1:**
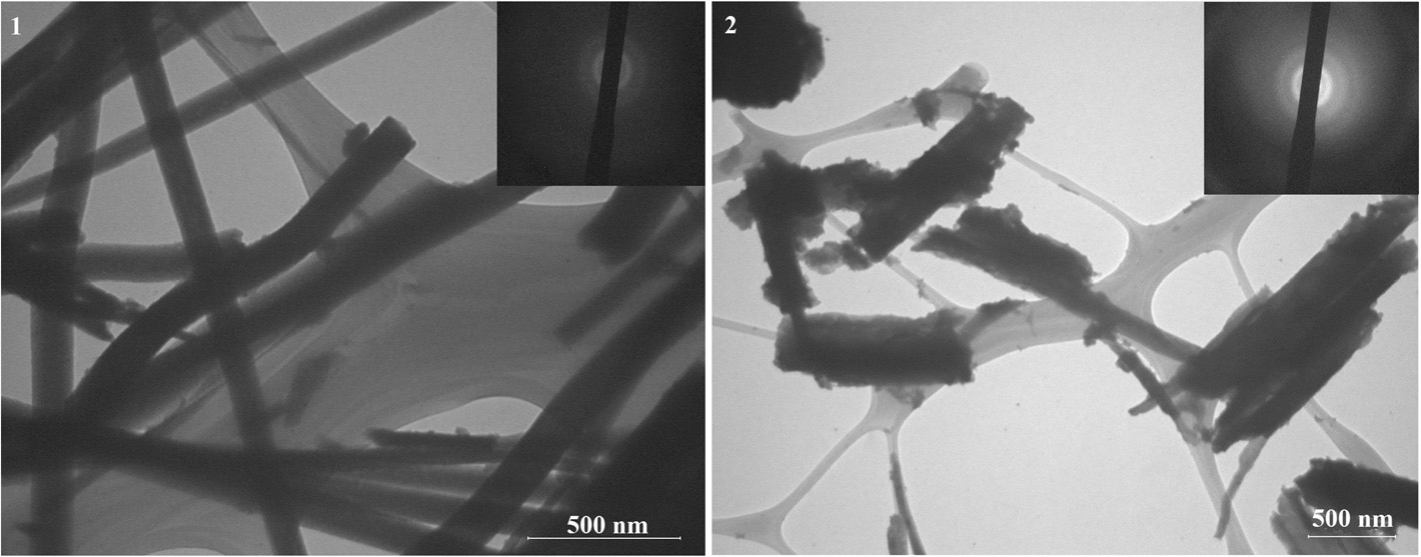
TEM images of sample A (1) and sample B (2) and corresponding SAED patterns

Figure 1 displays TEM images of the obtained tin(II) oxalate samples. The figure shows that sample A has a wire-like form, while particles of sample B have an unspecified form and are more porous. These differences in morphology are caused by various pH of tin(II) oxalate precipitation. Presented appropriate selected area electron diffraction (SAED) patterns of the samples shows that the particles are polycrystalline.

**Fig. 2:**
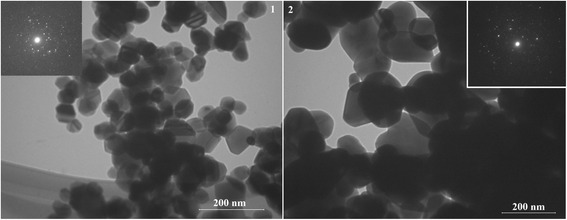
TEM images of sample 1 (1) and sample 4 (2) and corresponding SAED patterns

**Fig. 3:**
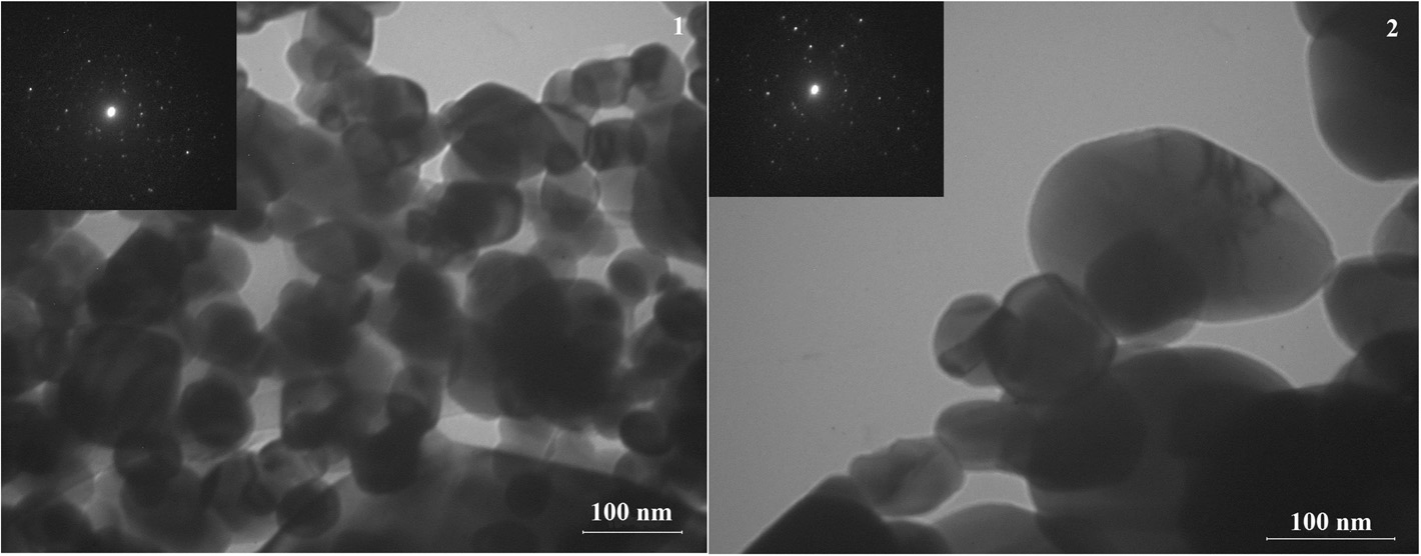
TEM images of sample 2 (1) and sample 5 (2) and corresponding SAED patterns

**Fig. 4:**
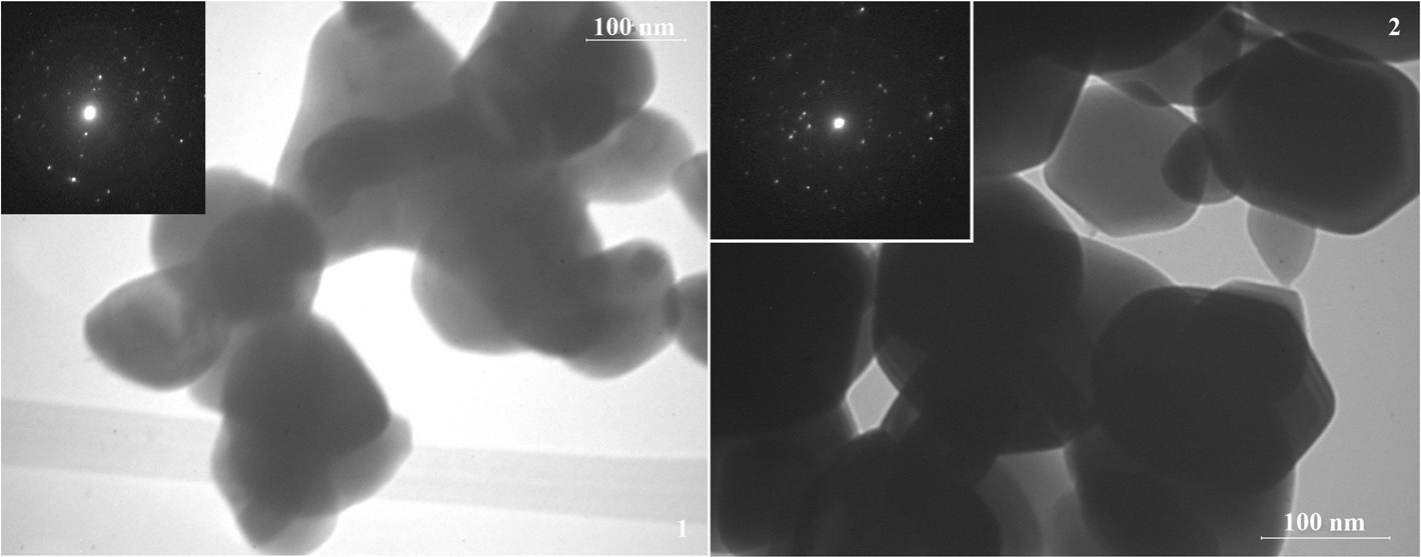
TEM images of sample 3 (1) and sample 6 (2) and corresponding SAED patterns

Figures 2, 3, and 4 show high-resolution TEM images of the synthesized samples of SnO_2_, in which the individual crystal sizes are in the range of 40–200 nm. The TEM images also show that the particles of the SnO_2_ samples obtained from sample A are more agglomerated, characterized by smaller size (average size is 60–80 nm), and have are more uncertain form. While the particle size of the SnO_2_ samples obtained from sample B reached 200 nm. Powders represented as individual particles that have a pronounced hexagonal shape, which is especially distinct for sample 6. Thus, to obtain better crystals of tin(IV), oxide preferably used ammonium oxalate as a precursor of tin(II) oxalate.

Presented SAED patterns of the samples demonstrate single-crystalline spots. And, it will allow obtaining sensitive materials with high values of sensor response. Since the crystal quality does not seem to be good, the development of synthesis technique for improving the quality of the single crystals is therefore necessary.

### X-ray Diffraction

XRD (X-ray diffraction) measurements were conducted using X-ray diffractometer Ultima IV Rigaku with CuКa radiation.

**Fig. 5:**
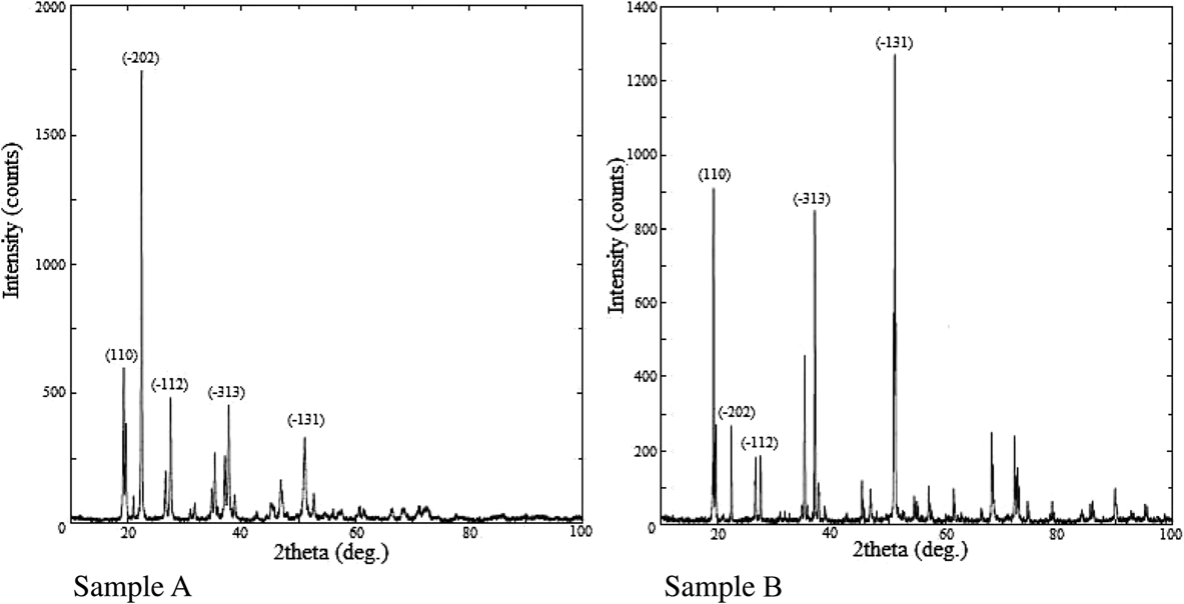
The XRD patterns of SnC_2_O_4_

Figure 5 shows XRD spectra of the obtained samples of tin(II) oxalate which fit to the pure tin(II) oxalate (according card no. 01-072-9689, PDF-2/Release 2011 RD, ICDD). Diffraction patterns have different intensities of the main peaks. These differences are due to different morphology of samples A and B.

The average crystallite sizes of the obtained samples of SnC_2_O_4_ are 45.7 and 67.9 nm, respectively. The structural parameters (crystal grain size, lattice constants) of SnC_2_O_4_ samples presented in Table 3.

**Table 3:** The structural parameters of SnC_2_O_4_ samples

Sample	hkl	2*θ*, deg.	Glancing angle *d*, nm	Crystallite size, nm	Lattice constant, nm		
					*a*	*b*	*c*
Sample A	110	19.26	0.46058	45.7	1.035	0.549	0.821
	−202	22.30	0.39671				
	−112	27.51	0.32391				
	−313	37.71	0.23834				
	−131	51.11	0.17858				
Sample B	110	19.29	0.45074	67.9	1.033	0.548	0.820
	−202	22.44	0.39596				
	−112	27.57	0.32330				
	−313	37.06	0.24239				
	−131	51.12	0.17855				

**Fig. 6:**
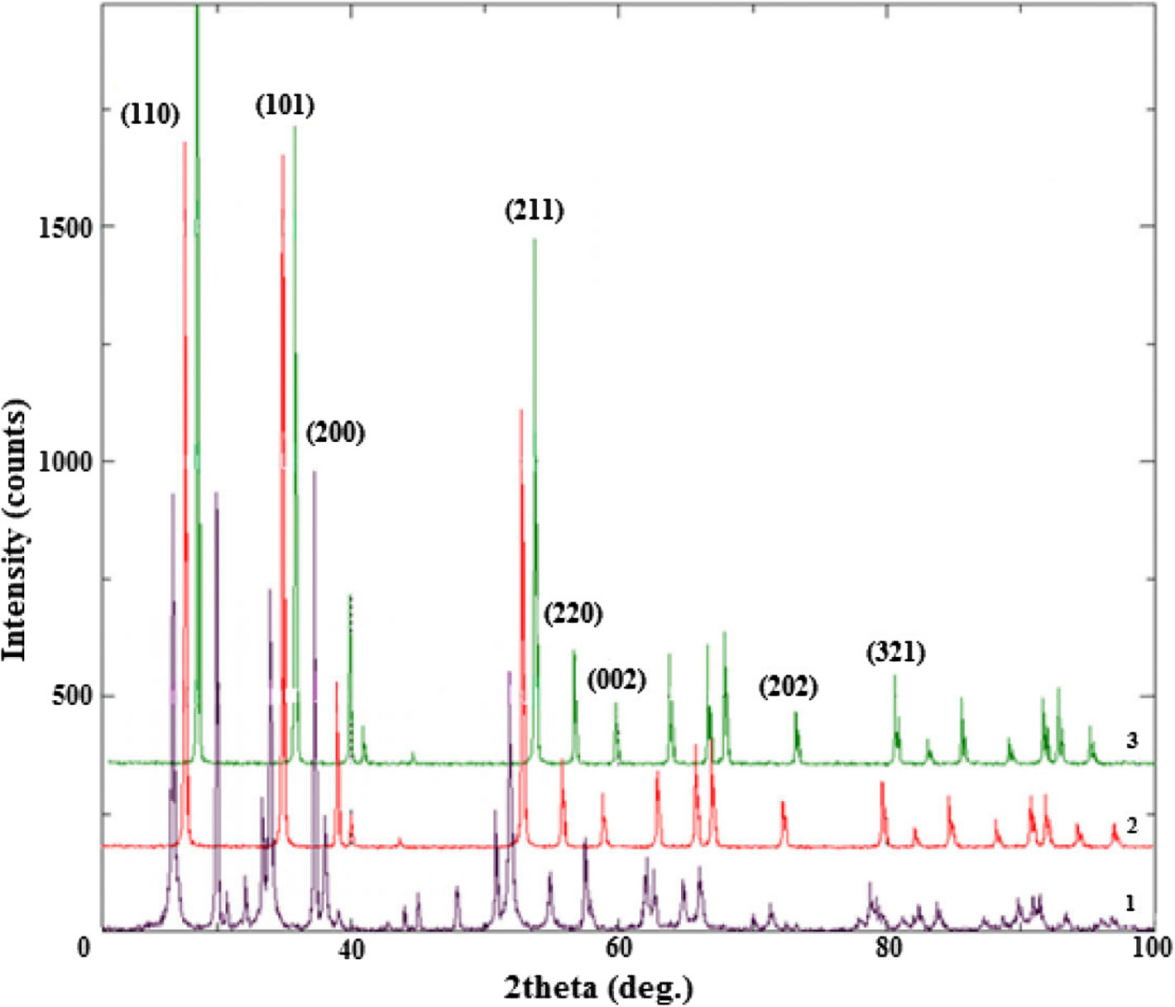
The XRD patterns of SnO_2_: *1* – sample 1; *2*– sample 2; *3* – sample 3

**Fig. 7:**
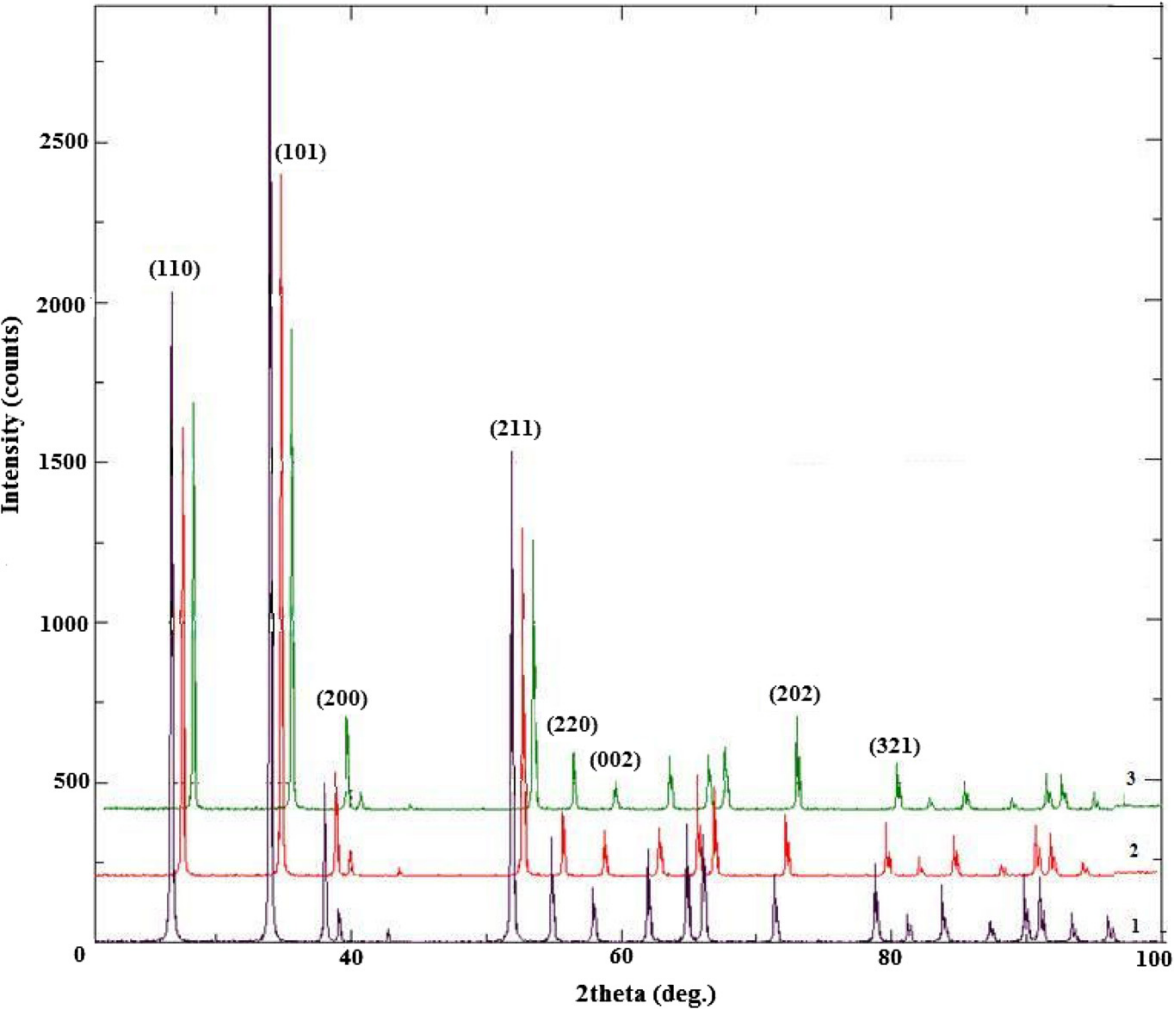
The XRD patterns of SnO_2_: *1* – sample 4; *2*– sample 5; *3* – sample 6

X-ray diffraction of the samples, which were obtained by decomposition of tin(II) oxalate at different temperatures, shows that pure SnO_2_ is formed in all cases besides powder obtained from sample A at temperature 850 K. Thus, 950 K is the minimum temperature for tin(IV) oxide synthesis from oxalate (Figs. 6, 7). Most distinct peaks on XRD patterns correspond to (110), (101) and (211) crystal faces (according card no. 1000062, USER (COD)). All diffraction lines can be indexed to the tetragonal rutile phase. For the samples, which were obtained from sample A the most distinct peak is (110), while for the samples, which were obtained from sample B, peak (101), that indicates the beginning growth of 1D nanostructures [21].

The comparison of the defined lattice constants for the samples with their standard values (*a* = 0.476, *p* = 0.318) shows that the crystalline lattice of SnO_2_ in samples was not deformed.

The structural parameters of SnO_2_ samples are presented in Table 4. According to the data presented in the table, with increasing temperature of heat treatment, the average crystallite size (and therefore particle size) increases. This is caused by the process of sintering particles.

**Table 4:** The structural parameters of SnO_2_ samples

Sample	hkl	2*θ*, deg.	Glancing angle *d*, nm	Crystallite size, nm	Lattice constant, nm	
					*a*	*c*
Sample 1	110	26.59	0.33494	45.3	0.474	0.319
	101	33.90	0.26419			
	211	51.79	0.17637			
Sample 2	110	26.59	0.33490	64.7	0.474	0.319
	101	33.90	0.26419			
	211	51.81	0.17632			
Sample 3	110	26.59	0.33502	80.7	0.474	0.319
	101	33.90	0.26425			
	211	51.79	0.17637			
Sample 4	110	26.64	0.33437	57.8	0.474	0.319
	101	33.94	0.26391			
	211	51.85	0.17620			
Sample 5	110	26.67	0.33402	73.6	0.474	0.319
	101	33.94	0.26393			
	211	51.84	0.17621			
Sample 6	110	26.60	0.33490	74.3	0.474	0.319
	101	33.91	0.26415			
	211	51.78	0.17641			

### Optical Spectra

Bandgap of SnO_2_ samples was determined by measuring the optical absorption of SnO_2_ films. Measurements were performed on a spectrophotometer UV-5800 PC.

**Fig. 8:**
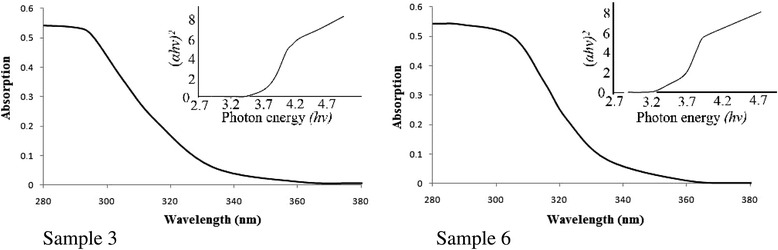
Dependences of the absorption coefficient on wavelength

Figure 8 shows dependences of the absorption coefficient on wavelength for samples 3 and 6. Limit wavelength values determined from the obtained diagrams are 328 and 336 nm for samples 3 and 6, respectively. The bandgap was calculated by the formula:

where *h* is the Planck constant and c is the speed of light.

The absorption coefficient *α* can also be expressed as:

$$ {\left(\alpha h\nu \right)}^2\infty \left(h\nu -\varDelta E\right), $$

where hν is the photon energy. Plots of (*αhν)*^*2*^ versus *hν* can be derived from the absorption data in Fig. 8 as shown in the inset.

The values of the bandgap for samples 3 and 6 are different and equal to 3.78 and 3.69 eV, respectively These data show that the precursor affects not only the morphology of particles SnO_2_ samples, but also their electrical properties.

## Conclusions

Characteristics of tin(IV) oxide highly depend on precursors used for the synthesis of tin(II) oxalate, which is confirmed by studies of electron microscopy, X-ray analysis, and optical spectra. The individual crystal sizes of synthesized SnO_2_ samples are in the range of 40–200 nm. The crystal lattice of SnO_2_ samples had shown no significant singular deformations. It is typical for the beginning growth of 1D nanostructures for the samples, which were obtained using tin chloride(II) and ammonium oxalate.
